# How Effective Is Auditory–Verbal Therapy (AVT) for Building Language Development of Children with Cochlear Implants? A Systematic Review

**DOI:** 10.3390/life11030239

**Published:** 2021-03-13

**Authors:** Paris Binos, Elina Nirgianaki, George Psillas

**Affiliations:** 1 Department of Rehabilitation Sciences, Cyprus University of Technology, 3036 Limassol, Cyprus; 2 Department of Speech & Language Therapy, University of Peloponnese, 22131 Tripoli, Greece; elinanirgianaki@gmail.com; 3 1st Academic ENT Department, AHEPA Hospital, Aristotle University of Thessaloniki, 54636 Thessaloniki, Greece; gpsyllas@auth.gr

**Keywords:** AVT, cochlear implants, speech, language, effectiveness

## Abstract

This systematic review sheds light on the effectiveness of auditory–verbal therapy (AVT) outcomes in children with cochlear implants (CIs). The presented outcome is based on research findings from the last 10 years. The systematic review was designed based on the Preferred Reporting Items for Systematic Reviews and Meta-Analyses (PRISMA) guidelines and Critical Appraisal of Treatment Evidence (CATE) checklist. Specific keywords were chosen based on the research question and searched on the PubMed database. All searched papers were analysed based on specific exclusion criteria and classified into four evidence levels. The results revealed that children who participated in AV therapy can achieve linguistic skills at the same level as their hearing peers. Voice quality seemed positively affected, placing young children with CIs in the normal range for receptive vocabulary development. In contrast, reading skills seemed less benefited. AV therapy seems to contribute to integration into mainstream society. Despite the recorded speech and language improvements of young children with CIs, the aim of AV therapy is still not fulfilled. AV therapy can be seen as the best clinical practice for young children with CIs till now, but the lack of well-controlled studies is undermining.

## 1. Introduction

Speech and language development are severely affected when hearing impairment (HI) is present [1]. The primary concern of caregivers of infants who receive cochlear implants (CIs) is how their infants will develop spoken language. According to the Centers for Disease Control and Prevention [2], annually, hearing loss is the most prevalent newborn congenital disorder, with 3 of 1000 children born with hearing loss (HL). On-time identification is only the first step. Children with HL will become effective communicators only when they start receiving early intervention that is identified as appropriate for HL children and implemented as evidence-based practices (EBPs) [3]. This is because HL children did not receive the same language feedback as their normal-hearing (NH) peers due to a lack of auditory input. The quality of the intervention services following the early identification will affect the communication outcomes [4]. The aim of habilitation programs is for children with HL to function similarly as their hearing peers. Recent advancements in cochlear implant technology have conveyed research interest in auditory-driven approaches for HL children, such as auditory–verbal therapy (AVT) [5].

AVT is a listening and spoken language (LSL) instructional approach. The AG Bell Academy for Listening and Spoken Language provides certification to specialists who deal with HL children [6]. Therefore, for caregivers who would like their HL children to develop speech, AVT can be seen as a reliable solution [7]. The aim of AVT is that HL children must learn to use hearing as the main sensory modality in developing speech production skills, since these children manage the listening experiences created by AVT [8,9]. Hearing deprivation affects all aspects of language development and affects children diagnosed with language delay when school age begins [10,11]. Today, despite the implementation of a newborn hearing screening, which has decreased late diagnosis, there are still late diagnoses during the school age years. Another aim of AVT is to achieve full integration into mainstream society following an array of 10 principles of clinical practice.

These 10 principles aim to trigger the auditory cortex of these HL children and help communication partners to benefit their children’s receptive and expressive language skills [12]. The skill to learn spoken language is seriously affected after 4 years of auditory deprivation since the brain plasticity gradually decreases through age [1]. Usually, children with HL are diagnosed immediately after their birth due to newborn hearing screening, and they gain access to sound input. Research has shown that identification and habilitation before 6 months of age are linked with improved communication skills during development [13].

Social participation is based heavily on language and many factors affecting the success in communication of CI children. Factors like the age at implantation, other disorders, duration of deafness, residual hearing before the operation, early diagnosis and habilitation, parent’s feedback, auditory skills, and mainly two language options support communication in deaf or children with HL [14]. In Reference [15], during postsurgery period, children with HL were candidates for two main habilitation modes that dominated in the literature. The first choice was spoken/oral language, and the other option was manual based. The newborn screening programs and the provision of early habilitation programs among identified children made oral language the most selected option of families and caregivers, at least for more than 90% of cases who had hearing parents [16]. The choice of sign language (manual based) concerns mainly the other 10% of children who have deaf parents, or they do not have the requirements to develop oral language. However, Punch and Hyde [17] underlined the need of parents to get more informed on communication mode options as well as schooling options to support their selection. Aside from the two basic approaches, there are also some alternatives that combine techniques and share slightly different methodologies with them. Some are visual based (sign language), and others are auditory driven (AVT and AV therapies). There are also programs that combine both main approaches (total communication, bilingual/bicultural) to achieve maximum results in communication.

As such, aural/oral programs promote the use of residual hearing. They also promote speech reading techniques to develop spoken language. On the other hand, the total communication approach is based on the use of sign language and auditory and oral techniques. Audition is crucial for AVT, and residual hearing is a necessary component for acquisition of spoken language without the use of additional cues (speech reading and gestures). Here, the caregiver is an important component at each session, and the habilitation goals are designed based on individual needs and possibilities of both the child and the caregiver [18]. As natural as possible, environments are used to develop listening and talking skills [19]. Despite the agreement that AVT promotes spoken language in children with HL [20], evidence-informed practice for AVT has not been explored during the last 10 years.

Research found in the PubMed database in last 10 years has concluded that there are some serious effects of AVT on crucial aspects of the linguistic skills of CI children. These recorded effects are classified into five key areas: receptive and expressive language, reading comprehension, speech intelligibility, social interaction, and receptive vocabulary [19,21,22,23,24,25]. Based on the Preferred Reporting Items for Systematic Reviews and Meta-Analyses (PRISMA) guidelines, the research question of the current systematic review is this:

“What is the effectiveness of AVT in the speech and language development of children with cochlear implants?”

## 2. Materials and Methods

### 2.1. Identification

The present methodological framework is based on the Preferred Reporting Items for Systematic Reviews and Meta-Analyses (PRISMA) guidelines to ensure replicability and transparency [26]. PRISMA guidelines provide an evidence-based guide for reporting in systematic reviews. The present systematic search is based on the database of PubMed, including only “full text” peer-reviewed articles, and was conducted in December 2020. The quality of the studies was evaluated using the Critical Appraisal of Treatment Evidence (CATE) checklist [27,28]. Each of the above-presented studies was evidenced and analysed by CATE levels. CATE levels were classified from highest/most credible (Ia) to lowest/least credible (IV). Thus, level Ia applies to a well-designed meta-analysis of >1 randomized controlled trials (RCTs), level Ib is only about RCTs, level IIa includes well-designed controlled studies with no randomization. Level IIb is assigned to well-designed semi-experimental studies, level III is for well-designed nonexperimental studies (correlation and case studies), and level IV includes only expert reports, conference outcomes, and clinical reports of respected authorities [29].

### 2.2. Screening

#### 2.2.1. Inclusion Criteria

The search process was based on the inclusion of specific keywords, such as “auditory verbal therapy—AND—cochlear implant.” Peer-reviewed papers were considered eligible if they were published only in the last decade in English and concerned children at birth up to 17 years. The search included all types of studies, such as original articles, clinical trial phases I and II, classical articles, randomized controlled trials, and comparative studies, and all had to be available as “full texts.” The study located published peer-reviewed studies in the last decade onwards. There were data published before the selected decade, but the present hearing technology is not similar to the CI technology a decade prior. This systematic review discussed only studies that deal with the effects of AVT on auditory perception and expressive and receptive language.

#### 2.2.2. Exclusion Criteria

The present search strategy excluded papers concerning adult population or nonhumans. Similarly, any reviews or papers not published in peer-reviewed journals were excluded. Studies with a different aim other than to shed light on the effectiveness of AVT were also excluded. Other papers were excluded if they had studied the effectiveness of the AVT approach but not with CI recipients. The search strategy also excluded papers about the effectiveness of AVT in families but not in communication. More studies were excluded if their participants received AVT but the effectiveness of the approach was not studied.

The initial search for the term “auditory verbal therapy” gave 2364 articles from 1951 to 2020. The use of the term “cochlear implant” decreased the outcome to 204, while the time restriction for the inclusion of papers published in the last decade resulted in 132 articles. The total number of 8 articles was based on the aforementioned exclusion criteria.

## 3. Results

### 3.1. Literature Outcome

**Figure 1:**
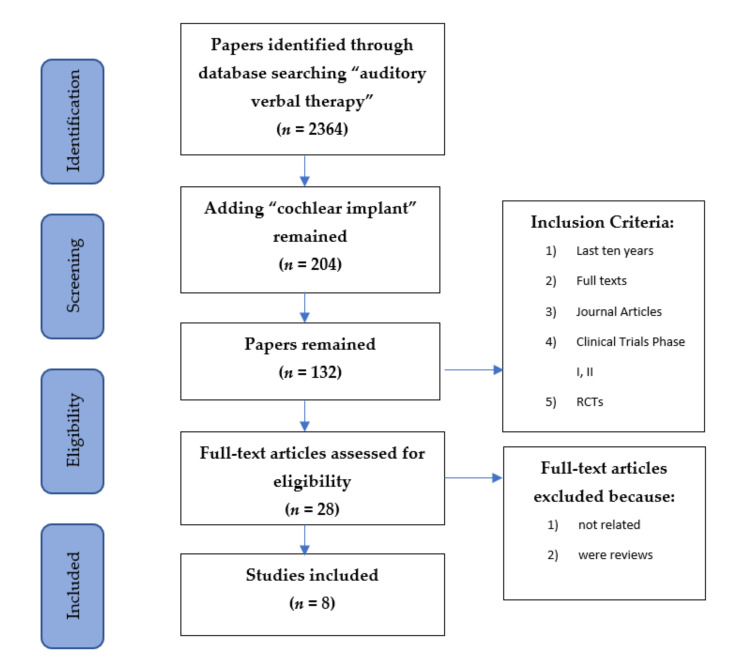
Flow chart of reviewed papers with exclusion and inclusion criteria.

The literature search yielded 2364 records by using the keyword “auditory verbal therapy.” At this stage, another keyword (cochlear implant) was added, and 204 hits remained. After screening all titles and abstracts and implementing the present inclusion criteria, 132 hits remained. The remaining full-text articles were assessed for eligibility, and only 28 papers were further analysed. At this stage, 20 papers were excluded as they were not relevant to the aim of the present systematic review or they were reviews, and the final remaining papers were 8 (Figure 1).

To address the research question, the findings of these eight studies are presented below. Table 1 shows details of the studies investigating the effectiveness of auditory–verbal therapy (AVT) on the speech and language development of children with cochlear implants. Results of CATE analysis and evidence level of the studies included in the review are presented in Table 2.

### 3.2. Investigating Studies

First, the studies reveal that there is an important progression in terms of both language development and auditory comprehension among children with CI who receive AVT; however, they are not always catching up with their NH peers [19,23].

Jackson and Schatschneider [19] conducted a longitudinal case series study to illustrate the progress of the expressive and receptive language performance of children with HI enrolled in AVT. The inclusion criteria included consistent use of sensory devices and parents who spoke English only with no other conditions. Participation in the program was over a period covering at least two testing points equivalent to at least 1 year. Eleven participants (46%) were using cochlear implants, and 13 (54%) other aids, while the mean age of diagnosis was 10 months. The degree of HL differed and ranged from mild to profound in the better ear, with most participants having median to great HL. Participants with cochlear implants had severe or profound hearing loss prior to implantation and were tested at 6-month intervals. There were two testing time points. The first was 79% at a minimum of three points, 67% at four, 58% at five, 29% at six, and 12% at nine or more. Raw and standard scores on both receptive and expressive subtests were recorded, while a re-evaluation of the files took place to ensure the accuracy of the file records.

The results revealed significant differences in the group as a whole. The affected areas included expressive language raw scores over time. Relative to the norms for age-matched hearing peers, HL children demonstrated improved performance but not similar to that of the normal-hearing group. With regard to auditory comprehension, the caseload as a group showed a significant change over time; however, the participants were not closing the gap with their normal-hearing peers. Moreover, the duration of habilitation was the key factor explaining the recorded differences. Many factors negatively affected the outcome of the research. The small unbalanced sample size, the heterogeneous nature of the sample, the degree of hearing loss, the age of identification, and the duration of hearing aid use were the limitations of this study.

Percy-Smith et al. [23] conducted a longitudinal comparative study to compare the early vocabulary development of three groups of participants: children with hearing loss (HL) wearing either CI or HA/Bahs in comparison with their NH peers. All HL children were enrolled in a 3-year AV program. All children had bilateral HI and moderate HL (i.e., 41–70 dB). Children were tested and assessed with standardized communication tests. The median age of diagnosis and intervention for the two groups did not differ significantly (i.e., 6 months for diagnosis and 13 and 12 months for intervention with hearing technology). The educational status of caregivers was similar in both groups, as well as the spread of diagnoses. The findings revealed no significant difference between the groups.

Although the results revealed no recorded statistically significant difference, children with HI progressed over the 3-year period. The scores of the NH group in years 1 and 2 were significantly different compared with those of the children with HI. In year 3, testing was not statistically different. The small group size; its heterogeneity in most aspects, such as age at diagnosis, age at intervention, and aetiology; the variability of therapy sessions; differences in HTL between the two groups of HI children; the inclusion of children with additional disabilities; and no control group of normal-hearing children constitute the limitations of this study.

Several studies employ a comparative design for children with CI who receive AVT and those taking other habilitative approaches, such as auditory–oral (AO) and bilingual–bicultural (BB) [21], standard habilitation [22], oral communication (OC) and total communication (TC) [24], and AO and sign and spoken language (SS) [25], most of them revealing higher language scores among children receiving AVT.

Dettman et al. [21] conducted a study to investigate the effectiveness of a habilitation approach on speech perception and language skills. Eight children enrolled in AV, 23 in AO, and 8 in bilingual–bicultural (BB) therapy participated in the study. The inclusion criteria were congenital severe to profound or profound hearing loss, no other disorder or cognitive delay, no abnormal cochleae or other unusual findings, and cochlear implantation before 4 years of age. The results showed that the AV group was better than the BB group in all measures of speech perception and performed better than the AO group in the Peaboby Picture Vocabulary Test (PPVT) age equivalent in years and CNC word scores. The group also reached the highest mean score in CNC words for phonemes and words; the AV and AO groups performed equally well in Bamford-Kowal-Bench (BKB) sentences and better than the BB group. However, the AV group was significantly older at age of testing and also at hearing age than both the AO and BB groups. Communication mode was not a significant factor of outcomes when the participants were individually matched, but the small sample size reduced the power of this comparison. The study did not include a control group of normal-hearing children; did not match the children for socioeconomic status, maternal education level, or parental involvement; and could not control for test age and duration post-implantation, which may have affected some results.

Percy-Smith et al. [22] compared the language level of 94 children with cochlear implants who had received Danish standard habilitation with that of 36 children with cochlear implants who had received AV practice. The median ages of the participants were 47 and 48 months, respectively, for the two cohorts, and the total median age of hearing with CI was 24 months. The participants received standard habilitation after a minimum of 6 months with CI, with most of them (72.5%) having 1–2 hours of habilitation weekly and 44% of their parents having participated in the sessions. Children who had received AV therapy were with 100% parent participation. The homogeneity of the sample was secured by the duration of AV therapy (2 years) and the commitment of the participants to follow this program.

Fisher’s exact tests were used to analyse the data. The estimated odds ratios and confidence intervals were based on Wald tests, while the *p*-values for covariates with more than two levels were based on likelihood-ratio tests. The participants who had received AV therapy scored significantly higher in all three speech and language tests, while no other covariate (i.e., gender) affected the result as much as type of intervention. It was unknown to AVT experts whether the participants had any other disability and what the rate of familiarity was that the participants developed.

Thomas and Zwolan [24] conducted a retrospective longitudinal study that evaluated the effect of communication mode on spoken language in children with cochlear implants over a 7-year period in six different phases (i.e., yearly from 2 to 7 years post-implantation). All the participants fulfilled the U.S. Food and Drug Administration’s (FDA) criteria as CI candidates and were diagnosed with a bilateral sensorineural HL. Children received their CI before the age of 5 years, and the aetiology of deafness was unknown. All the participants had been consistently using an auditory–verbal (AV), oral communication (OC), or total communication (TC) habilitative approach. The mean ages at CI activation were 19.74 months for the AV group, 32.29 months for the OC group, and 40.9 months for the TC group. All groups differed significantly in terms of socioeconomic status, with 10% of Medicaid for the AV group, 23% for the OC group, and 38% for the TC group.

Speech and language, reading, and speech intelligibility scores were evaluated through a linear mixed model as an effect of communication mode. Α confirmatory analysis supposed that individuals enrolled in the AV group would outperform members of the OC and TC groups in terms of their speech language, intelligibility, and reading scores. The results indicated significantly higher scores for the children in the AV group than both those in the OC and TC groups in every measure and at every test interval (*p* < 0.05). Compared with the AV score, the differences ranged from 0.2% to 21.2% for the OC group and from 18% to 39.2% for the TC group. Some of the limitations of this study include lack of a control group of children with normal hearing, lack of reliability measures, and differences among the three groups in age at implant and socioeconomic status which may have contributed to the findings.

In the study of Yanbay et al. [25], 42 children using similar levels of implant technology were split into three groups according to therapy program (i.e., auditory–oral (AO) (14), aural–verbal (AV) (18), and sign and spoken language (SS) (10) programs) in order for the language outcomes among the three groups to be investigated. Moreover, the relationships between language skills and other factors (e.g., gender, preimplant hearing age, age at hearing aid fitting, age at enrolment into the communication program, and socioeconomic status) were examined.

The sample was selected following many inclusion criteria: among others, the diagnosis of bilateral severe sensorineural hearing loss identified by 12 months of age, CI implant before the age of 4 years, and enrolment in the selected communication program for at least 10 months. PPVT revealed no significant difference across the three groups, although a significant relationship between the test and caregiver role was evident. A significant effect of family involvement and socioeconomic status was found, but no significant difference was revealed across the three groups in postimplant Preschool Language Scale-4 (PLS-4) auditory comprehension (AC) scores. Almost half of the SS children, most of the AO children, and just over half of the AVT children had PLS-4 expressive communication (EC) scores equal to or higher than the standard score of 85. Large variability within groups, relatively small sample size and data, clinicians not blinded to children’s language outcomes, and no comparison with a normal-hearing control group constitute some of the limitations of this study.

Finally, two studies stress the effectiveness of AVT in association with early identification of HL and intervention [30,31], revealing language capabilities within normal limits (WNL) among children with CI who attain this combination.

In the study of Fulcher et al. [30], children who were early identified (EI) with hearing loss (HL) were compared with children who were late identified (LI). The inclusion criteria were HL diagnosis, enrolment in AVT, and the amplification provided before 12 months of age. On the other hand, LI participants were included in the study if they were identified to be with HL and provided with amplification and AVT after 12 months and before 5 years of age. The two groups did not differ significantly in terms of gender, SES, and maternal education levels. Speech and language skills were evaluated at ages 3, 4, and 5 years. The results for both groups (EI and LI) were reviewed and rescored to ensure accuracy of test information (interjudge reliability was 94.7%, and intrajudge reliability 99.2%).

The EI group was better than the LI group in all speech, receptive vocabulary, and receptive/expressive language measures at all ages and in all levels of HI severity, while the differences were increasing as the age was increasing. The results suggested that the performance within normal limits (WNL) was not only possible for the majority of EI children enrolled in AVT but also can be achieved at as early as 3 years of age. The study did not include a normal-hearing control group.

Sahli [31] investigated the development of 169 children with profound bilateral sensorineural hearing loss. All participants received a single-sided CI, and none of them had any known/diagnosed disability. The average chronological age was 26.4 months (ranging between 18 and 36 months). The participants were divided into three groups according to the age of diagnosis, age of amplification, and starting age of therapy. Twenty-one of them started AVT before 6 months, 84 between 6 and 12 months, and 64 between 12 and 18 months. The test was conducted on the 1st, 3rd, 6th, 9th, 12th, 18th, 24th, and 36th months of the follow-up. Of the children who started AVT before 6 months, 90.5% had “normal” language development, while the percentages of “normal” language development were 77.4% and 75% for children who started AVT between 6 and 12 and between 12 and 18 months, respectively. The rate of “delay” in development was found to be increasing as the starting age of training was delayed (i.e., children with “delayed” language development were 9.5% of those who started AVT before 6 months, 22.6% of those who started between 6 and 12 months, and 25.0% of the children who started 12–18 months. Lack of an NH control group and reliability measures, as well variable characteristics within the groups, are some of the limitations of this study.

## 4. Discussion

The present systematic review was based only on the PubMed database. The search revealed increased research interest in developing habilitation strategies for children with hearing impairment. The limited evidence on the effectiveness of AVT is enriched with new data, and this is one of the most important clinical implications this study brings. Future counselling for parents who want their infants with HI to acquire and develop spoken language and speech pathologists who provide the service should take into account these new research outcomes.

Our data analysis classified the findings into three main areas. The first area concerned the recorded progression in terms of both language development and auditory comprehension among children with CI who received AVT [19,23]. The second area of classification concerned language skills that benefited more from the participation of CI children in AVT programs instead of other habilitative approaches (i.e., AO, BB, OC, TC) [21,22,24,25]. The last area of data classification concerned the role of early identification of HL and intervention. CI children who received AVT in association with both parameters have had language capabilities within normal limits (WNL) [30,31].

Although recent evidence from the published papers that were reviewed revealed the positive role of AVT with regard to the speech and language skills of CI children, it is still difficult to generalize. AVT can be seen as a tool for CI children to catch up with their NH peers, but future studies need to clarify many weaknesses in the process of evidence and validity. Similar to studies before 2010 [32], participation in AVT still needs broader inclusion criteria and should involve participants of any performance instead of the best performers. Future experimental studies should aim to include well-controlled studies with larger heterogeneous samples of children with CI. Another limitation concerning all the presented studies is that none of the eight had used randomization to create contrasting conditions, and some did not present important findings [21,23], or the findings were not statistically significant [25]. On the other hand, significant similarities with studies before 2010 were also found. A number of the analysed reviewed studies [19,22,24] concluded similarly or partly similarly [30] that AVT benefited receptive and expressive language skills [33,34].

To sum up, there is strong evidence of the effectiveness of AVT on the development of all linguistic skills but moderate or weak evidence on receptive vocabulary skills and use of BKB sentences or CNC words [21]. The review brought to light that the best linguistic skills can only be measured in infants diagnosed before the first 6 months of age [31]. This specific group of participants was the most probable to “catch-up” with their normal-hearing peers.

## 5. Conclusions

This systematic review reported the outcomes of eight peer-reviewed studies based on the PubMed database on the effectiveness of AVT in the communication skills of children with cochlear implants. Auditory–verbal therapy (AVT) can be seen as an instructional approach suitable for HL children. To our knowledge, this is the latest systematic review of research published over the last 10 years about the effectiveness of AVT.

Retrospective and longitudinal studies revealed statistically significant improvements in both expressive language and auditory comprehension based on AVT. There is also a lack of well-controlled studies addressing the use of AVT. On the other hand, a lack of these studies does not prevent us from concluding that AVT is an effective intervention in the habilitation of children with cochlear implants based on the aforementioned data. AVT has already been proved to be an effective approach for these specific individuals with cochlear implants.

## Figures and Tables

**Table 1:** Details of studies investigating the effectiveness of auditory–verbal therapy (AVT) on the speech and language development of children with cochlear implants.

Study	Language	Design	Aim	Participants	Examined Areas of Language	Tools	Outcome
Percy-Smith et al. (2018) [23]	Danish	Longitudinal, comparative (3 years)	Early vocabulary development among children with CI compared with children with HA/Bahs (all enrolled in a 3-year AV program)	36—CI, 19—HA	Receptive and productive vocabulary, language understanding	PPVT-4, Reynell, Viborg materialet	Children with HI progressed over a 3-year period, but they did not reach the same level as children with NH
Jackson and Schatschneider (2014) [19]	English	Longitudinal	The rate of language development of HI children in a private clinical intervention program, receiving weekly AVT	12 boys, 12 girls	Expressive language, auditory comprehension	PLS-4	Within-subjects comparisons revealed statistically significant improvements (*p* < 0.05) in both expressive language and auditory comprehension
Thomas and Zwolan (2019) [24]	English	Retrospective, comparative	Effect of communication mode (AV, OC, TC) on spoken language	39—AV, 107—OC, 57—TC	Receptive and expressive language, speech intelligibility, reading	PPVT, EVT, WJPC, GFT-AAPS	Significantly higher scores for the AV group
Percy-Smith et al. (2017) [22]	Danish	Retrospective, comparative	Effect of (re)habilitation strategy on speech language for early cochlear-implanted children	94—SH, 36—AV	Receptive and productive vocabulary, language understanding	PPVT-4, Reynell, Viborg materialet	Children in AV intervention outperformed children in SH in all tests of speech and language
Yanbay et al. (2014) [25]	English	Retrospective cohort	Differences in outcomes among children with cochlear implants enrolled in AO, AVT, and SS programs	14—AO, 18—AV, 10—SS	Receptive vocabulary, auditory comprehension, expressive communication	PPVT, PLS-4, IRSAD, FPRS	No significant differences in language outcomes across the three groups
Dettman et al. (2013) [21]	English	Retrospective	The relative impact of early intervention approach on speech perception and language skills	8—AV, 23—AO, 8—BB	Receptive vocabulary, speech perception (correct phonemes and correct words)	PPVT, CNC words, BKB sentences	AV group showed the least receptive vocabulary delay and highest mean score in CNC words—AV and AO groups performed equally well in BKB sentences
Fulcher et al. (2012) [30]	English	Prospective/retrospective, comparative	Examine the speech/language outcomes of children with HL by comparing them with respect to (i) early versus later-identified HL and (ii) degree of hearing loss	45 early identified (12 months) and 49 late identified (>12 months)	Speech production, receptive vocabulary, receptive and expressive language	Goldman–Fristoe Test of Articulation-2; Sounds-in-Words (GFTA-2); PPVT-4, a receptive vocabulary measure; PLS-4	By 3 years of age, 93% of all early-identified participants scored within normal limits for speech, 90% for understanding vocabulary, and 95% for receptive and expressive language
Sahli (2019) [31]	Turkish	Retrospective	Effect of hearing loss on early childhood in terms of development, instrumentation/amplification, and the starting age of training	92 males, 77 females	Hearing, comprehension, and use of language, receptive, and expressive language capabilities	DDST-II	Children diagnosed before 6 months, instrumented between 3 and 6 months, and started with AVT revealed normal skills in their language capabilities

**Table 2:** Results of Critical Appraisal of Treatment Evidence (CATE) analysis and evidence level of the studies included in the review.

Aspects/Studies	Percy-Smith et al. (2018)	Jackson and Schatschneider (2014)	Thomas and Zwolan (2019)	Percy-Smith et al. (2017)	Yanbay et al. (2014)	Dettman et al. (2013)	Fulcher et al. (2012)	Sahli (2019)
Plausible rationale for the study?	Yes	Yes	Yes	Yes	Yes	Yes	Yes	Yes
Was the evidence from an experimental study?	No	No	Yes	Yes	Yes	Yes	Yes	No
Was there a control group or condition?	Yes	No	Yes	Yes	Yes	Yes	Yes	No
Was randomization used to create contrasting conditions?	No	No	No	No	No	No	No	No
Were methods and participants specified prospectively?	Yes	Yes	Yes	Yes	Yes	Yes	Yes	Yes
Were patients recognizable at the beginning and end?	Yes	Yes	Yes	Yes	Yes	Yes	Yes	Yes
Was treatment described clearly and implemented as intended?	No	Yes	No	Yes	Yes	Yes	Yes	No
Was the measure valid and reliable?	Yes	Yes	Yes	Yes	Yes	Yes	Yes	Yes
Was the outcome evaluated with blinding?	Νο	No	No	Νο	No	No	No	No
Nuisance variables	See text	See text	See text	See text	See text	See text	See text	See text
Was the finding statistically significant?	No	Yes	Yes	Yes	No	Partly	Yes	Yes
If the finding was not statistically significant, was statistical power adequate?	No	N/A	Ν/Α ^1^	N/A	Yes	No	N/A	N/A
Was the finding important?	No	Yes	Yes	Yes	Yes	No	Yes	Yes
Was the finding precise? ^2^	No	No	Yes	Yes	Yes	Yes	No	No
Was there a substantial cost–benefit advantage?	UR	UR	UR ^3^	UR	UR	UR	UR	UR
Evidence level	IIb	IIb	IIa	IIa	IIa	IIa	IIa	IIb

^1^ Not Applicable. ^2^ Precise if confidence intervals were reported. ^3^ Unable to rate this criterion with the information provided.
